# Impact of COVID-19 vaccination on risk perception: a cross- sectional study on vaccinated people: Valentina De Nicolò

**DOI:** 10.1093/eurpub/ckac129.664

**Published:** 2022-10-25

**Authors:** V De Nicolò, P Villari, C De Vito

**Affiliations:** Department of Public Health, Sapienza University, Rome, Italy

## Abstract

**Background:**

High risk perception and perceived threat of COVID-19 had played an important role on public attitudes toward vaccination and protective countermeasures acting as motivational factors to perform behaviors that facilitated disease prevention. To explore COVID-19 vaccination influence we conducted a cross-sectional study on vaccinated people (18-80 years old).

**Methods:**

Partecipants, randomly selected, were recruited during the vaccination campaign in the Hub of Cosenza, Calabria Region, Italy, at the end of the 2nd wave of pandemic (Jul-Aug 2021). A multivariable logistic regression model was built to calculate odds ratios (OR) and 95% confidence intervals (95% CI) comparing risk perception and preventive measures confidence pre vs. post vaccination.

**Results:**

Globally 625 partecipants fully responded; 51.4% women, mean age 40.5 years (SD +/-15.36). Infection risk perception and protective measures adherence significantly decreased after the vaccination even if a significant gender gap was present; women were always more worried and respectful than men. More prudent partecipants had a significantly higher mean age. 64.2% of participants believed that compliance with social distancing was yet absolutely necessary after the immunization and about half of them believed that also the use of masks was yet necessary. Results of multivariable analysis confirmed that risk perception decreases after vaccination in different contexts: workplace (0.65; 95% CI 0.44-0.94); sport activities (0.60; 95% CI 0.39-0.95); bars and restaurants (0.51; 95% CI 0.33-0.80); means of transport (0.32; 95% CI 0.19-0.50) as well as handwashing practice (2.23 95% CI 1.20-4.12).

**Conclusions:**

The research shows that COVID-19 vaccination significantly decreases self-perceived risk and adherence to preventive measures. Public Health communication strategies could underline that COVID-19 vaccination is indispensabile but not sufficient to protect the World against this devastating catastrophe.

**Key messages:**

• Public Health should promote consciousness and strengthen the importance of health-protective measures in order to further reduce risk of human-to-human transmission after immunization.

• Even after vaccination, extra precautions are still required and necessary to do not nullify vaccination protective effect, mostly in more exposed and less careful groups like young people.

